# THE SOCIAL COST OF MODIFIABLE RISK FACTORS IN SINGAPORE

**DOI:** 10.1093/geroni/igac059.3021

**Published:** 2022-12-20

**Authors:** Vanessa Tan, Stefan Ma, Cynthia Chen

**Affiliations:** National University of Singapore, Singapore, Singapore; Ministry of Health Singapore, Singapore, Singapore; National University of Singapore, Singapore, Singapore

## Abstract

Close to half of total disease burdens are attributable to modifiable risk factors, indicating that many illnesses are preventable by modifying behaviours such as increasing physical activity levels or maintaining a healthy diet. This study measures the social costs attributable to modifiable risks in Singapore, one of the most rapidly ageing populations in the world. Measuring the social cost of modifiable risk factors can help public health policymakers prioritise public health programmes and allocate resources. Our study builds on the comparative risk assessment framework from the Global Burden of Disease study. We used a prevalence-based cost-of-illness approach to estimate the social cost attributable to modifiable risk factors. We included healthcare costs from inpatient hospitalisation and productivity losses from absenteeism and premature mortality. Our results found that metabolic risks had the highest social cost of S$2.20 billion in 2019, followed by lifestyle risks of S$1.98 billion and substance risks of S$1.56 billion. Across the risk factors, the social costs were largely driven by productivity losses, heavily skewed towards the older working-age group. For metabolic risks, approximately 80% of the total cost (S$1.82 billion) was from those aged above 45 years old. This study provides evidence of the high social cost of modifiable risks and highlights the importance of developing holistic public health promotion programmes. Our findings suggest that implementing effective population-based programmes targeting multiple modifiable risks would have a strong potential to manage rising disease burdens and healthcare costs, especially with an ageing population.

